# Associations between *dru* Types and SCC*mec* Cassettes

**DOI:** 10.1371/journal.pone.0061860

**Published:** 2013-04-25

**Authors:** Mette D. Bartels, Kit Boye, Duarte C. Oliveira, Peder Worning, Richard Goering, Henrik Westh

**Affiliations:** 1 Department of Clinical Microbiology, Hvidovre Hospital, Hvidovre, Denmark; 2 Faculty of Health and Medical Sciences, University of Copenhagen, Copenhagen, Denmark; 3 Centro de Recursos Microbiológicos (CREM), Faculdade de Ciências e Tecnologia, Univ. Nova de Lisboa, Caparica, Portugal; 4 Instituto de Tecnologia Química e Biológica (ITQB), Univ. Nova de Lisboa, Oeiras, Portugal; 5 Department of Medical Microbiology and Immunology, Creighton University School of Medicine, Omaha, Nebraska, United States of America; University of Calgary, Canada

## Abstract

Molecular typing is an important tool in the investigation of methicillin resistant *Staphylococcus aureus* (MRSA) outbreaks and in following the evolution of MRSA. The staphylococcal cassette chromosome *mec* (SCC*mec*) contains a hypervariable region with a variable number of 40 bp repeats named direct repeat units (*dru*). The *dru* region has been suggested as a supplementary typing method for MRSA and an international nomenclature exists. The purpose of this study was to investigate the diversity and variability of the *dru* region in a diverse collection of MRSA. We studied 302 MRSA isolates harbouring SCC*mec* types I to VI. The isolates represented a broad genetic background based on Staphylococcal protein A (*spa*) typing and multilocus sequence typing (MLST) and included 68 isolates (68 patients) from an outbreak with t024-ST8-IVa and 26 isolates from the same patient. Sequencing identified 53 *dru* types (dt) in 283 isolates, while eighteen isolates contained no *dru* repeats and one isolate resisted sequencing. The most common *dru* type, dt10a, was present in 53% of the sequenced isolates and was found in all SCC*mec* types, except type II. Seven (10%) of the 68 epidemiologically related patients had isolates with *dru* type variants indicating that *dru* typing is not useful as a first line epidemiological typing tool. However, MRSA isolates cultured from a single patient over a three year period exhibited a single *dru* type. The finding of dt10a in most SCC*mec* types suggests that *dru* and *mec*A originate from the same Staphylococcus species.

## Introduction

Our understanding of MRSA evolution and outbreaks has substantially increased as sequence-based typing methods have been more commonly used [1]. Multilocus sequence typing (MLST) is preferred for evolutionary studies, but is time consuming and expensive. Sequencing of the Staphylococcal protein A (*spa*) gene is a commonly used sequence-based typing method for local MRSA outbreak investigations and has excellent interlaboratory reproducibility [2], [3]. Typing of the staphylococcal cassette chromosome *mec* (SCC*mec*) adds further information and is predominantly done by PCR fragment analysis [4]–[6] but sequence-based typing methods based on structures in the SCC*mec*, such as *ccr*B typing and *dru* typing have been introduced [7], [8]. SCC*mec* typing, when combined with MLST and/or *spa* typing, is central in MRSA typing as it allows identification of international MRSA clones [1], [9].

In 1991, Ryffel *et al* [10] described the region between IS*431mec* and the *mec*A gene in SCC*mec*. This region was named the hypervariable region (HVR) due to DNA length polymorphisms. Ryffel and colleagues sequenced the HVR of one MRSA strain and identified ten direct repeat units (*dru*) of 40 bp each. Most initial studies compared the gel band sizes or hybridization patterns of *dru* PCR products [11]–[16] while a few smaller studies sequenced the *dru* region [17], [18]. The sequencing of the *dru* region of 277 EMRSA-15 and EMRSA-16 led to a universal nomenclature [7], [19] and since then many new *dru* types have been reported [20]–[22]; www.dru-typing.org.

We studied the *dru* region of 302 MRSA isolates of global geographic distribution representing a broad range of genetic backgrounds and the SCC*mec* types I to VI. The collection included sixty-eight isolates from epidemiologically related Danish patients with t024-ST8-IVa and 26 isolates over a four-year period from the same patient. The purpose of our study was to investigate the diversity of the *dru* region in relation to different SCC*mec* cassettes and to evaluate its variability in both an epidemiological outbreak and over time in the same patient.

## Materials and Methods

### MRSA Isolates

Three-hundred and two MRSA isolates were included. Seventy-three of the isolates were from the International collection of Herminia de Lencastre and 229 were isolated in Denmark. The collection contained 65 *spa* types. The isolates harboured SCC*mec* types I to VI plus several SCC*mec* subtypes (Table 1). Only one isolate per patient was included except for one patient where 26 isolates, cultured from May 2005 to November 2009, were examined for longitudinal dru diversity. To test variability of the *dru* region during a seven year long MRSA outbreak we included 68 isolates of the same *spa* and SCC*mec* type from 68 patients that were epidemiologically linked.

**Table 1:** SCC*mec* type of the 302 isolates.

SCC*mec* type	Number of isolates
I	14
IA	5
I-VAR	1
II	17
III	6
IIIA	11
IIIB	1
IV^a^	15
IVa	136
IVb	1
IVc	28
IVd	3
IVE	1
IVg	5
IVh	7
IV-NT^b^	1
V	35
VI	3
NT^c^	12
	302

a as not been subtyped.

b Subtyping gave no result.

c non-typeable.

Typing: All isolates were *spa* typed and SCC*mec* typed as previously described [4]. Some SCC*mec* type IV, type I and type III isolates from the International collection were subtyped [6], [23]. Multilocus sequence typing (MLST) was available on all the International isolates and was performed on at least one isolate of each *spa* type in the Danish collection [24]. For *dru* PCR, the primers HVR1∶5′ ACTATTCCCTCAGGCGTCC 3′ and HVR2∶5′ GGAGTTAATCTACGTCTCATC 3′ were used [11]. Isolates that were *dru* PCR negative by this primerset were tested with an additional two primer sets to determine whether the *dru* region had been deleted:

*dru*F2∶5′CACATTAATCGCACTTTTATTTCCA3’and *dru*R2∶5′CTTGCCTAGGGGTATGGCTC 3′ and *dru*GF: 5′GTTAGCATATTACCTCTCCTTGC3’and *dru*GR 5′GCCGATTGTGCTTGATGAG 3′ [7]. In one *dru* PCR negative isolate the region between *mec*A and IS*431* was sequenced using the following primers: 5′CGGCTACAGTGATAACATCC 3′and 5′TCCAGATTACAACTTCACCAGG 3′. All *dru* PCR products were enzymatically purified as previously described [25] and sequenced on both strands using the same primers as in the primary PCR. Sequences were analyzed and aligned using Chromas Pro (Technelysium Pty, Australia). New repeats were confirmed by resequencing. *dru* repeats (dr, 40 bps) and *dru* types (dt, the combination of *dru* repeats) were named according to the nomenclature published by Goering *et al* [7], www.dru-typing.org. For some isolates the *dru* region was established based on an ongoing whole genome sequencing project on an Illumina HiSeq platform.

To compare the relatedness between *dru* types, the TRST plug-in tool of BioNumerics v7.0 was used for cluster analyses. With this plug-in, sequences are compared and aligned using an algorithm based on the DSI (duplication, substitution, and indels) model for pairwise alignment of repeats, which considers that modification of sequences can occur through duplication of tandem repeats, substitutions, insertions, and deletions (the latter two events are collectively termed indels) (1). A similarity matrix is generated based on the DSI model and used to construct a minimum spanning tree (MST); the type with the greatest number of related types is assigned as the root node, and the other types derive from this node. In the present study, the default parameters were used for alignment of sequences. The software creates groups of certain distance intervals or similarity values (which BioNumerics terms bins) and converts the data into distance units. Because of the highly clonal nature of the MRSA isolates investigated in the present study, the bin distance was set to 0.5%, (i.e., the distance between two entries with 99.5 to 100% similarity was set at 0) on the MST, and the distance between two entries with 99 to <99.5% similarity was 1. Using the MSTs, *dru* types were deemed to belong to different subgroups if they were separated by an MST distance of ≥2 (i.e., if they showed ≤98.5% similarity). Therefore, if two *dru* types were at an MST distance of <2, they were considered to be closely related (i.e.,they formed a subgroup).

## Results

**Figure 1:**
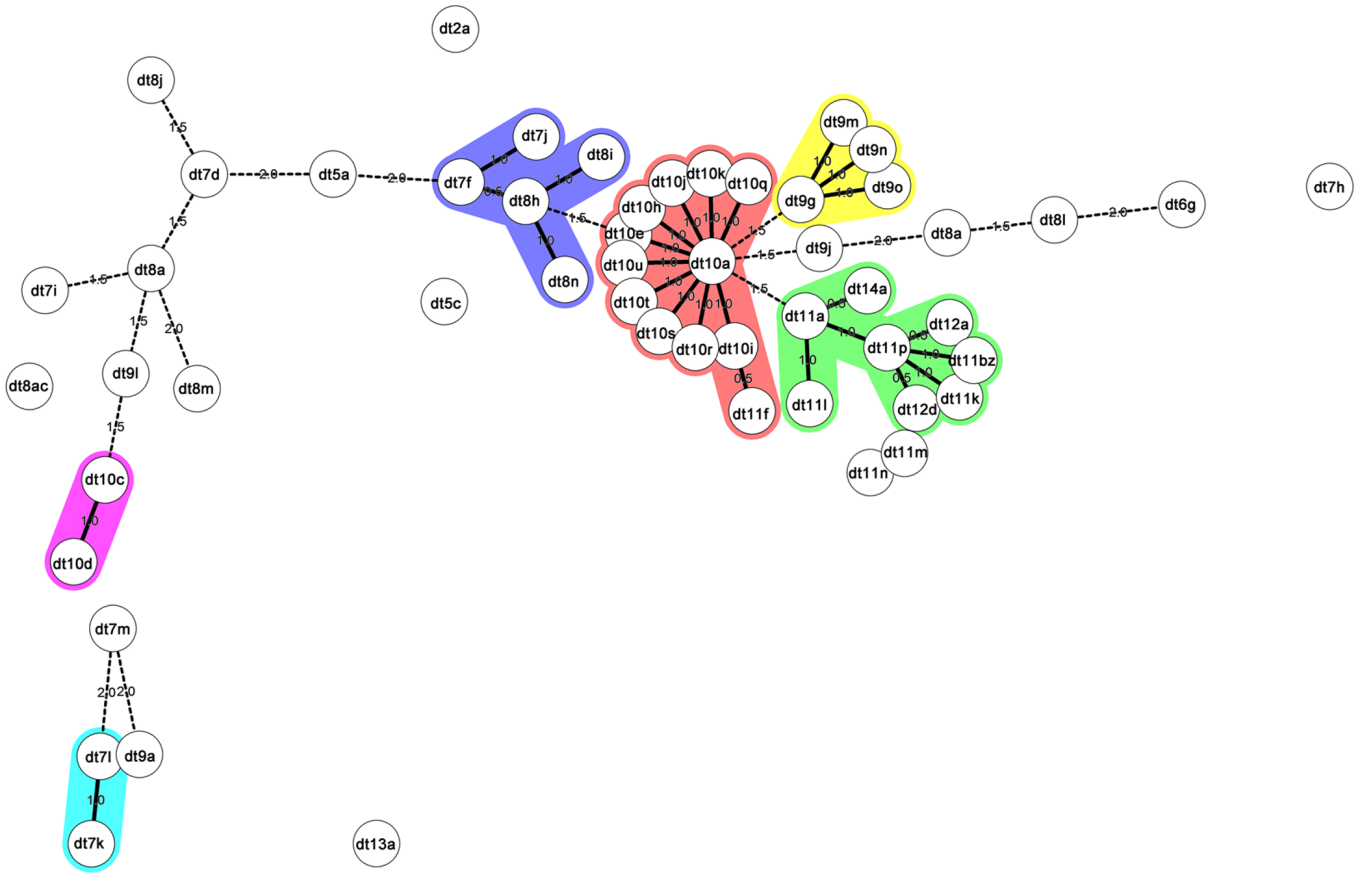
Minimum Spanning Tree generated using the BioNumerics software program representing the 53 *dru* types observed in the study isolates. Numerical values on the branches indicate the similarity (MST distance) between different *dru* types. BioNumerics software creates similarity values (termed bins) and converts these data into distance units. The bin unit distance was set to 0.5% (i.e., *dru* types at a distance of 1 on the MST have between 99 and 99.5% similarity, types at a distance of 2 have between 98.5 and 99% similarity, etc.). The *dru* types were assigned to the same (colored) cluster if they were separated by an MST distance of <2 (i.e., if they showed >98.5% similarity).

Two-hundred and eighty-three of the 302 isolates (94%) had a *dru* type and were all identified by the first primer set. Additionally one isolate was PCR positive by the first primer set but resisted sequencing. Seventeen isolates were *dru* negative by all three primer sets and one isolate had a double band with the primer set *dru*GF/*dru*GR but when sequenced contained no *dru* repeats. The 19 *dru* sequence negative isolates harboured SCC*mec* I (6 isolates), SCC*mec* II (11 isolates), SCC*mec* IV (1 isolate) and one isolate had lost part of SCC*mec* when retrieved from the −80°C freezer. In one *dru* PCR negative isolate (*spa* type t003, SCC*mec* II), the region between the *mec*A gene and IS*431* was sequenced and contained no *dru* repeats. Among the 283 *dru* sequenced isolates we found 53 *dru* types. The *dru* types contained from two to 14 repeats with the majority of *dru* types having 10 repeats (13 sequence variants) (Figure 1). The most common *dru* type, dt10a, was found in 53% (161) of the sequenced isolates and identified in all SCC*mec* types except type II. In Figure 1, dt10a is compared to the other 52 dts. When excluding the SCC*mec* non-typeable (NT) isolates, forty-five *dru* types were restricted to one SCC*mec* type. However, 36 of these *dru* types were only found in one or two isolates each. The remaining nine *dru* types were found in three to 14 isolates with two to five *spa* types in each group belonging to two or three different CCs (Table 2). Seven *dru* types were found in at least two SCC*mec* types when excluding the non-typeable cassettes (Table 3).

**Table 2:** *dru* types found in more than two isolates and restricted to one SCC*mec* type.

*dru* type	SCC*mec* type^a^	*spa* types (ST/CC)	Number of isolates
dt9g	IVa	t008 (8/8)	14
dt11p	V	t008 (8/8), t024 (8/8), t084 (15/15), t292 (8/8), t127 (748/1)	11
dt11a	V	t018 (36/30), t024 (8/8), t123 (45/45), t591 (−/−)	7
dt9a	II	t002 (5/5), t018 (36/30)	5
dt8l	IVa	t024 (8/8), t126 (72/−)	5
dt7l	III	t037 (239/8), t421 (239/8)	5
dt10u	IVc	t1798 (88/−), t019 (30/30), t975 (30/30)	4
dt7i	IVa	t127 (1/1), t024 (8/8)	3
dt8h	I	t001 (228/5), t041 (−/−)	3

a SCC*mec* non-typeable isolates are excluded.

**Table 3:** *dru* types present in more than one SCC*mec* type^a^.

*dru* type	SCC*mec* types	*spa* types (ST/CC)	Number of isolates
dt10a	I, I-VAR, IIIA, IVa, IVb, IVc,	30 different *spa* types	160
	IVd, IVg, IVh,V,VI		
dt7f	I, IIIA, IVa, IVh	t008(8/8), t459(239/8), t128(1/1),	6
		t036(8/8), t009(254/−)	
dt7j	IVa, IVc	t128(1/1), t008(8/8)	5
dt9n	IVa, IVc	t127(1/1), t044(80/80)	3
dt10d	IA, IIIA	t051(247/8), t037(239/8)	2
dt10h	IVh, V	t022(22/22), t002(5/5)	2
dt10t	IVa, IVc	t186 (88/−), t376 (−/−)	2
Total			181

a SCC*mec* non-typeable isolates are excluded.

The patient with 26 MRSA isolates over a four-year period had her first MRSA isolated in May 2005, where we found different *spa* types and *dru* types in five samples taken on the same day: t024-SCC*mec*V-dt11p (throat and groin), t024-V-dru negative (wound), t024-V-dt8l (axilla) and t292-V-dt11p (axilla). In October 2005 a t024-V-dt11bz was isolated from her groin. Just one month later, t024-IVa-dt10a was found in three samples and continued to be the type cultured from the patient throughout 2006 (seven samples), 2007 (one sample), 2008 (three samples) and 2009 (three samples), except for one sample from January 2006 that was similar to the October 2005 t024-V-dt11bz isolate.

To test the variability of the *dru* region in an outbreak situation, 68 epidemiologically related isolates of *spa* type t024-IVa from 68 patients were analyzed. These patients had either been admitted to the same hospital at the same time or lived in the same nursing homes. Sixty-one of the 68 isolates had dt10a, while the remaining seven isolates had dt2a (1), 7h (1), 7i (2), 8l (3). Two of the dt 8l and dt7h were isolated in 2004, dt2a in 2005, the two dt7i isolates in 2006 and one dt8l in 2008. Within the same nursing home three different dts were found (10a,7h and 8l).

## Discussion

In this study we found 53 *dru* types in 281 MRSA isolates suggesting a high diversity in the *dru* region. Thirty-three new *dru* types were found. We believe that the main reason we identified so many new *dru* types was that our collection was selected to be very diverse in regard to genetic background, SCC*mec* types and geography. Identical SCC*mec* types were often found to have different *dru* types and the same *dru* type was sometimes found in different SCC*mec* types. A single *dru* type, dt10a, dominated (53%) both in the Danish and International isolates as has been observed by others [7], [19], [20]. The Danish isolates in this study originated from the years 2003 to 2010 and we found dt10a in the entire period. The International isolates were collected over several decades and 45% had dt10a (33 of 73). We propose that this *dru* type is the ancestor *dru* type for most MRSA lineages and that the other *dru* types have evolved from it as is depicted in Figure 1. However, the lack of dt10a in SCC*mec*II isolates and the strain specific association of some *dru*-SCC*mec* types (e.g., the majority of t008 SCC*mec*IVa USA300 isolates are dt9g) seen here and elsewhere [7], [26], [27] are interesting exceptions. A blast search of staphylococcal genomic sequences reveals that dt10a is widespread in different MRSA strains including the archaic MRSA strain COL from 1965 and MRSA ST398 associated with livestock [28]. Methicillin resistant coagulase-negative staphylococci (CNS) have also been shown to carry dt10a [26] although additional studies comparing the *dru* types of human CNS to MRSA would be of interest. The frequent association of dt10a with the different SCC*mec* types included in this study supports evolution of the *dru* region after the insertion of the SCC*mec* into MSSA. However, because the *dru* region and the insertion sequence IS*431* are in close proximity within SCC*mec* it is possible that they may jointly insert downstream of *mecA* during the SCC*mec* assembly process. In addition, the stable association of *dru* types and SCC*mec ccrB* sequences has yielded interesting information regarding the potential movement of SCC*mec* elements in staphylococcal populations and MRSA phylogenetic interrelationships [26], [29].

In studies on EMRSA-15 and EMRSA-16 *dru* typing was useful to distinguish between subtypes of the same clone [7], [19], [20]. In the present study, seven patients had four *dru* types (dt2a, 7h,7i and 8l)) other than dt10a among 68 isolates from 68 epidemiologically related patients with MRSA t024-IVa. While these minor *dru* types are not closely related to dt10a based on MST (Figure 1) we believe that all 68 patients had related MRSA based on outbreak epidemiology. The dt2a and dt7i isolates were found in patients that had all been admitted to the same hospital, but not concomitantly. Sixteen of the 68 isolates were included in a recent study by our group where variations in the J3 region of the Danish t024-ST8-SCC*mec* IVa clone were identified [30]. One dt10a isolate and the dt7h isolate were from two persons living in the same nursing home and these two isolates had identical J3 variations (*ccr*AB4 and the *arc* gene cluster within J3). One of the patients with a dt8l isolate lived in another nursing home together with a person with a dt10a isolate both of which exhibited an alternative J3 variation (the *arc* gene cluster but no *ccr*AB4). The presence of the same J3 variation in isolates with different *dru* types suggests their derivation from a common ancestor. While the separation of the J3 and *dru* regions by IS*431* suggests independent evolution of the two regions [30], one cannot rule out the possibility that J3 deletion may have also influenced *dru* repeat structure.

We studied 26 isolates over a four-year period from a patient who seemed to have acquired several MRSA types over time. In the first year, the patient had five t024-V isolates with either dt8l, dt11p or dt11bz, and one isolate with a t292-V-11p. t292 is one repeat shorter than t024 and we believe that a deletion in the *spa* region is the most likely explanation for this difference [31]. The finding of three different *dru* types at the same time in the same genetic background could reflect evolution in the *dru* sequence in the very rare t024-V clone. An alternative explanation would be multiple acquisitions of t024 MRSA with different *dru* types which is consistent with the observation, five months later, that the patient had acquired MRSA t024 with a SCC*mec* IVa instead of SCC*mec* V. The shift from t024-V to t024-IVa in this patient was probably caused by replacement of one strain with another rather than the excision of SCC*mec* V and insertion of SCC*mec* IV. We base this assumption on the fact that the patient was living in a nursing home with an outbreak of t024-IVa-dt10a. However, whole genome sequencing of the type IV and type V isolates reveal that they were closely related (data not shown) and therefore an excision/insertion event of the SCC*mec* cannot be ruled out. Over a four-year period we found 20 t024-IVa isolates from this patient of which 19 had dt10a and one dt11bz suggesting a stable *dru* region, however, the mutation rate in the *dru* region is unknown. From each positive sample we only typed one MRSA colony. Typing several colonies from the same specimen could have resulted in the finding of MRSA isolates with different *dru* types and it is therefore possible that the patient was colonized with a dt10a and dt11bz at the same time. Based on our findings we believe that it is impossible to determine when and if one *dru* type evolved from the other in cases where different *dru* types are found in otherwise identical isolates from different patients. Changes in the repeat region of the *spa* gene has been analyzed [31], [32] and the mutation rate in the *spa* region during long-term persistence has been reported to be one genetic change every 70 months [32]. If the mutation rate in the *dru* region is similar, it is not surprising that we did not find genetic changes in the dt10a of the patient.

We found no *dru* repeats in 18 isolates. *dru* negative isolates have also been found in other studies [7], [33]. Four of the 18 *dru* negative isolates belong to ST225. In a study by Nübel *et al* [34] a deletion of the *dru* region was found in all isolates belonging to ST225. This indicates a spread of one MRSA clone rather than several episodes of acquisition of an SCC*mec* lacking the *dru* region into the same genetic background.

In conclusion, we found no correlation between *dru* type and SCC*mec* type, *spa* type or Sequence type. Therefore *dru* typing is not a first line epidemiological typing method for MRSA. In our study, the *dru* region seems rather stable in an outbreak situation, although isolates from seven of 68 patients had variations in the *dru* region. Therefore, a change in *dru* type in otherwise identical isolates is not enough to separate patients in an outbreak situation, but might be informative for epidemiological subtyping. Furthermore, *dru* typing can add interesting information on the evolution of *SCCmec.* Although this study includes 52 *dru* types it is worth noting that the *dru* database (www.dru-typing.org) contains 421 dts as of February 2013 and the conclusions drawn in this study are therefore based on a subset of the total *dru* database.

Over a seven year outbreak the *dru* type was retained in 90% of isolates, but the same common *dru* type, dt10a, was also found in several unrelated isolates. The finding of dt10a in so many different SCC*mec* types suggests that the *dru* region and the *mec*A originate from the same *Staphylococcus* species, while SCC*mec* II might have a different evolutionary pathway.
